# Gender discrimination of veterinary students and its impact on career aspiration: A mixed methods approach

**DOI:** 10.1002/vro2.47

**Published:** 2022-11-01

**Authors:** Katie Freestone, John Remnant, Erica Gummery

**Affiliations:** ^1^ University of Nottingham School of Veterinary Medicine and Science Loughborough Leicestershire UK

## Abstract

**Introduction:**

As the veterinary profession has become feminised, gender discrimination and its effects have been documented in practicing veterinary surgeons. However, research on gender discrimination experienced by veterinary students and its effects on recruitment and retention remains limited. This study aimed to increase understanding of veterinary students’ experiences of gender discrimination and its impact on their career aspirations.

**Methods:**

A questionnaire including statements with Likert‐style response options and free‐text questions was distributed to students studying veterinary medicine and science at a UK veterinary school in September 2020 (28% response rate). Two focus groups were carried out following the questionnaire to gain a deeper insight into student experiences.

**Results:**

Gender discrimination in a veterinary setting had been experienced by 34% of respondents, the majority (77%) on animal husbandry placements. Female students were more likely to report that their experiences of gender discrimination affected their career aspirations. Seven themes were identified from both the questionnaire and focus group data: stereotyping of certain fields, gender inequality on placements, the lesbian, gay, bisexual, transgender, queer and intersex, plus (LGBTQI+) community, encouraging reporting behaviours, barriers to reporting, education and the placement allocation.

**Conclusions:**

This study highlighted that gender discrimination was prevalent during animal husbandry placements, although reporting was infrequent and perceived negatively by students. Recommendations on how veterinary schools and the wider veterinary profession can support veterinary students are made as an outcome of this work.

## INTRODUCTION

The veterinary profession in the UK is becoming increasingly feminised.^1^ One study reported that 61% of practicing veterinary surgeons in the UK were female and 75% of veterinary surgeons qualifying between 2010 and 2019 were female,^2^ compared with 55% of veterinary surgeons graduating in 1993 reported as female.^3^ Other reports suggest that despite the rapidly changing gender balance veterinary professionals are discriminated against because of their gender.^4^

Despite the increasing number of women in the UK veterinary profession, the number of women in director, principal and partner roles within practices in 2014 was significantly lower than the number of men in these roles.^3^ Women are underrepresented as RCVS specialists and fellows.^3^ Furthermore, women are proportionally underrepresented in veterinary academia,^5^, ^6^ less likely to be a senior author on a research paper; they are significantly underrepresented in surgical and production animal research.^7^ This is not exclusive to the veterinary profession; female authors are underrepresented in medical research,^8^ the reasons behind this underrepresentation are unclear. It has been proposed that a ‘Lack of Fit Framework’ considers gender stereotyping as central to discrimination in the workplace.^9^ Expectations based on stereotypes bias perceptions of individuals’ ability to perform a role and give rise to biased judgements.

Discrimination in the veterinary profession against legally described protected characteristics, including gender has been documented in the UK.^10^ Gender discrimination made up 44% of all incidents of discrimination, was the most likely to go unreported and occurred most often in younger respondents.^10^ There is a limited information on the prevalence or effects of gender discrimination on veterinary students and the impact it may have on career aspirations in the UK.

In addition to stereotypes, gender discrimination and inequality have been found to occur at university for both medical and veterinary students;^11^, ^12^, ^13^ also, throughout their careers.^4^ Educating students on how to identify gender discrimination is important so that they are able to identify it in the future and feel able to report it.^12^ Widespread anecdotal evidence of gender discrimination on extra‐mural studies (EMS) motivated the authors to conduct this work.

This study aimed to describe students’ experiences of gender discrimination and its effects on their career aspirations within university and during EMS with the following questions:

1. What are veterinary students’ experiences of gender discrimination?
2. Does gender discrimination affect the career aspirations of veterinary students?

A greater understanding of the experiences of veterinary students is important to better enable educators, support students and implement changes.

## METHODS

A mixed methods study was designed to allow for triangulation of more generalisable quantitative data in phase one with richer qualitative data in phase two, given the complexity of this topic.^14^ The pragmatism paradigm taken by the authors focuses on the problem to be researched and the consequences of the research^15^; it accepts that a mixed approach may better help in solving a research problem.^15^ Focus groups were selected based on their efficiency in gathering data from multiple people, while being stimulated by the comments of others. Focus groups may also utilise the interaction of the group in a setting in which participants feel empowered to speak out.^16^

Ethical approval was granted from the University of Nottingham, School of Veterinary Medicine and Science (reference 3198‐200706). A questionnaire was designed to explore topics informed by existing literature. The questionnaire was piloted for clarity by a convenience sample of veterinary students known to the authors. Participation in the study was voluntary and participants received information regarding data handling and that they could withdraw their data from the study at any time. All data were anonymised. Responses were collected between 25 September and 31 October 2020.

Phase one involved the administration of an online survey of a questionnaire containing categorical items, questions with Likert‐like response options and questions requiring free‐text responses.

Questionnaire measures are summarised in Table 1. Rating scales allow for quantitative analysis of frequencies combined with measurement of opinion; they are commonly used in questionnaires to measure attitudes because they are simple for participants to use.^17^ The questionnaire (Supporting Information S1) was made available to 930 students at the University of Nottingham's School of Veterinary Medicine and Science using Jisc Online Surveys (Bristol, UK, 2020) through email by the authors. The email was distributed to all students in years 1–5 of the veterinary programme to obtain a representative sample. The results from the questionnaire were used to inform the focus group scripts utilised in phase two of the study. The scripts were designed to obtain qualitative data to provide deeper insights into student experiences.^18^

**TABLE 1:** A summary of measures collected within the study questionnaire

Topic	Question type
Demographic data, including gender, vet school attended, year of study, ethnic group, age and postcode	Tick box and free text (age and postcode)
Career aspiration	Tick box
Gender discrimination statements, including presence in the profession, concern about discrimination, equal opportunities and impact of gender	Likert‐style items (five point, strongly disagree to strongly disagree, with neutral midpoint)
Gender discrimination exposure (experienced, witnessed and heard of) including where it was experienced, action taken, who the discrimination was carried out by and the form of discrimination	Tick box
Discrimination impact and actions, including reasons for not reporting, whether it would be reported in the future, what would help reduce gender discrimination and effect on career aspiration	Free text (reasons for not reporting), free text and tick box (reducing gender discrimination), Likert‐style item (effect on career aspiration—four‐point scale from ‘not at all’ to ‘the deciding factor’)

*Note*: The full survey is available in ‘Supporting Information S1’.

The questionnaire comprised three sections. Section one collected demographic information. Section two collected information on student perceptions of gender discrimination within the veterinary profession via five‐point Likert‐like responses. Section three focused on students’ experiences of gender discrimination. Students described the type of discrimination, frequency and location of incidences where they felt directly discriminated against and stated whether they took any action. Free‐text questions collected data on (a) what interventions students believed would be useful to reduce gender discrimination and (b) what students saw as barriers to them reporting incidents of gender discrimination. A combination of question formats was used to avoid participant fatigue. To aid respondents, a definition of gender discrimination was provided^13^ and examples were included from the Equality and Human Rights Commission.

In phase two, questionnaire participants who had indicated that they would be willing to take part in focus group discussions were contacted via email (*n* = 8). The respondents were invited to take part in two focus groups, each containing four participants. This convenience sample was designed to elicit richer data on these students’ experiences. Consent forms were sent out to the participants and returned via email. Focus group discussions took place virtually via Microsoft Teams (Microsoft, Redmond, WA, USA, 2017) due to the COVID‐19 pandemic. Each focus group discussion lasted for 30 min. They were recorded within Microsoft Teams and auto‐transcribed using Microsoft Stream (Microsoft). The auto‐transcribing was manually checked for mistakes and corrected.

### Data analysis

Phase one survey data were exported into Microsoft Excel (Microsoft, 2019) for initial descriptive analysis and charts were produced in Prism v9.3.1 (GraphPad, San Diego, CA, USA). Stacked bar charts were used to illustrate differences in responses between groups. Quantitative data were then exported to IBM SPSS Statistics for Windows Version 27 (IBM Corp., Armonk, NY, USA, 2020) and coded. Due to there being few respondents in all ethnic groups, other than white, these have been grouped together to avoid the identification of individuals and will be referred to as underrepresented ethnic minority group. Due to low numbers, students who identified as an ‘other’ gender identity were excluded from analyses and figures specifically relating to gender to protect anonymity. However, their responses were included within the rest of the analyses. As data were non‐normally distributed and ordinal, Mann–Whitney *U*‐tests were used to test for associations between (1) respondent gender and the impact of gender discrimination on their career aspirations; (2) the impact on career aspiration of each of having experienced, witnessed or heard about gender discrimination; (3) respondent gender and agreement that gender discrimination was present; and (4) respondent gender and agreement that gender discrimination was a concern; *p* < 0.05 was considered statistically significant.

Qualitative data from the free‐text questions in the phase one questionnaire and from the phase two focus groups were thematically analysed using NVivo Version 12 Pro (QSR International, Melbourne, Australia, 2018; available from: www.qsrinternational.com/nvivo‐qualitative‐data‐analysis‐software/home; accessed 4 Oct 2022).^19^ For the free‐text questionnaire data, a general inductive approach was used to identify codes, which were then grouped into overarching themes.^20^ For the focus group transcripts, a hybrid approach of inductive and deductive approaches was used in analysis. The codes and themes identified in the qualitative data by the principal investigator (K. F.) were verified by a secondary investigator (E. G.) and codes altered if there were any disagreements between the investigators. The codes found in the qualitative data were triangulated with the findings from the quantitative data,^21^ these were categorised as convergent or complementary.

### Reflexivity statement

The principal investigator (K. F.) who carried out the focus groups identifies as a white, heterosexual female and at the time of data collection was intercalating a PGCert programme in veterinary education between the fourth and fifth years of the undergraduate veterinary degree programme at Nottingham. An anti‐discriminatory stance was maintained during the focus groups; however, there may be influence from the researcher's personal views within the questions asked and within the qualitative data analysis. Two authors first analysed the data independently before a process of review.^22^

## RESULTS

### Quantitative analysis

Of the 262 questionnaire responses received (28% response rate), one respondent failed to complete any questions and so was removed. All other respondents answered all relevant demographic questions. Of the remaining data, 83% of respondents identified as female, 16% of respondents identified as male and 1% of respondents identified with another gender identity (see Supporting Information S2). The percentage of male respondents was similar to the male student population (19%) reported within UK veterinary schools.^23^

**FIGURE 1:**
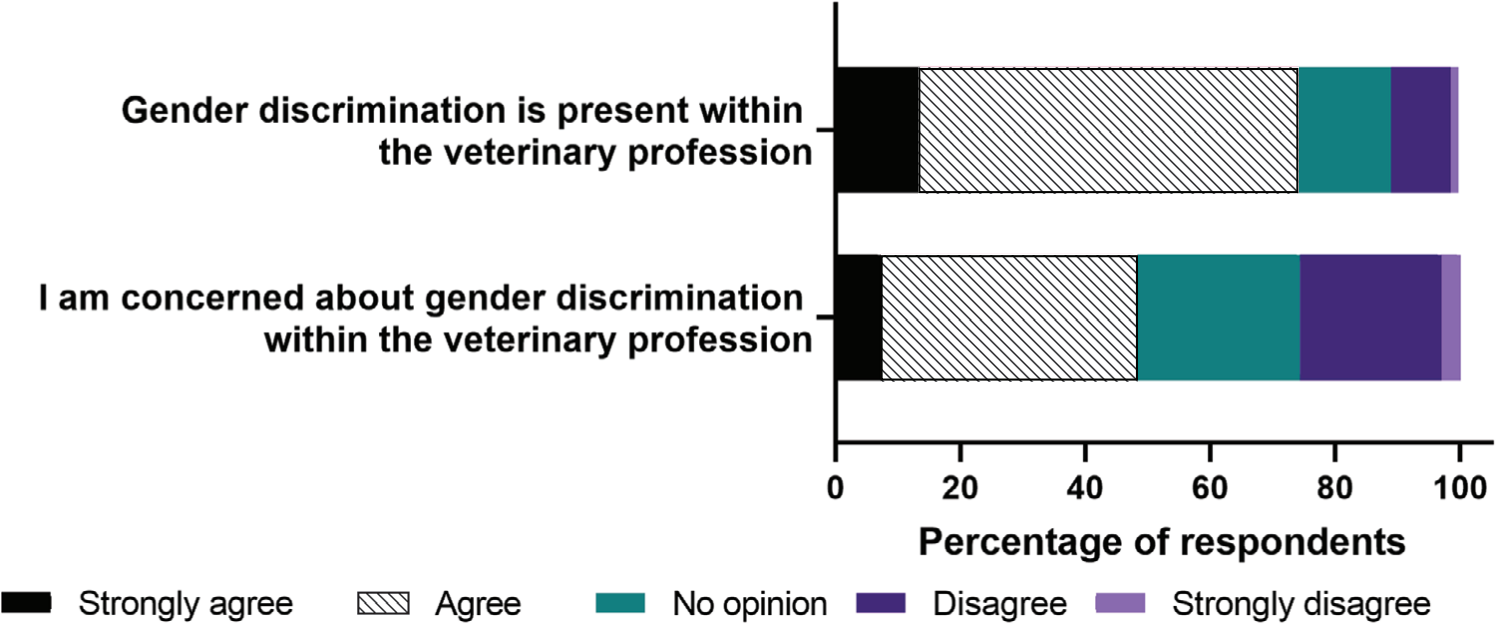
Level of agreement with statements about gender discrimination and the veterinary profession from a survey of undergraduate veterinary students (*n* = 262)

**FIGURE 2:**
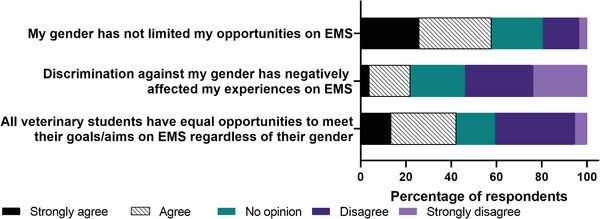
Level of agreement with statements about gender discrimination and extra‐mural studies (EMS) from a survey of undergraduate veterinary students (*n* = 262)

Figure 1 shows the majority (74%) of students either agreed or strongly agreed with the statement gender discrimination is present in the veterinary profession. Nearly half (48%) of respondents either strongly agreed or agreed that they were concerned about gender discrimination within the veterinary profession. There were no differences between male and female respondents relating to agreement that gender discrimination occurs (*p* = 0.13) and agreement that gender discrimination is of concern (*p* = 0.41). Figure 2 shows that regarding EMS (compulsory work experience mandated by the UK regulatory body for all veterinary students) including animal husbandry and clinical EMS, 42% of students agreed or strongly agreed that all veterinary students had equal opportunities to achieve their goals/aims on EMS. Just over half (57%) of respondents agreed or strongly agreed that their gender has not limited their opportunities on EMS. However, there was a difference of opinion between genders with 83% of male respondents and 53% of female students agreeing or strongly agreeing that their gender had not limited their opportunities on EMS.

When asked if they had experienced gender discrimination during EMS, 37% of female and 17% of male students responded they had. The majority (77%) of these students reported experiencing the discrimination on animal husbandry EMS. Of the students identifying as an underrepresented ethnic minority, 57% reported experiencing gender discrimination compared to 34% of all respondents.

Of students reporting having experienced gender discrimination, 64% said they never took action. Male students (86%) were less likely to take action than female students (61%). The most common type of gender discrimination experienced by students was verbal harassment (72%).

In relation to witnessing and hearing about other students experiencing gender discrimination in a veterinary setting (including EMS and veterinary school), 20% of respondents reported witnessing an incident and 70% of students reported hearing about an incident. Most incidents students witnessed or heard about were experienced on Animal Husbandry Extra Mural Studies (AHEMS).

**FIGURE 3:**
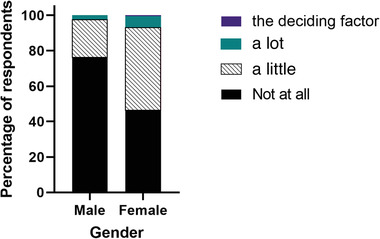
A graph showing the relationship between veterinary students’ gender identity and the extent to which their considerations and experiences of gender discrimination had affected their career aspirations on a four‐point scale (not at all, a little, a lot, the deciding factor) (*p* = 0.001)

**FIGURE 4:**
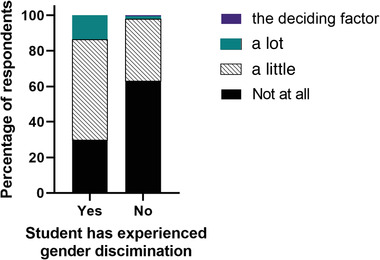
A graph showing the relationship between having (left) or having not (right) experienced gender discrimination and the extent to which their considerations and experiences of gender discrimination had affected their career aspirations on a four‐point scale (not at all, a little, a lot, the deciding factor) (*p* < 0.001)

When asked if experiences and considerations about gender discrimination had affected their career aspirations, 49% of respondents felt that their aspirations had been affected to some degree. Figure 3 shows how this differed between gender; female students were more likely to report that experiences and considerations around gender discrimination had affected their career aspirations either a little, a lot, or were the deciding factor (*p* = 0.001). Students who reported that they had experienced (*p* < 0.0001; shown in Figure 4), witnessed (*p* = 0.046) or heard about (*p* < 0.0001) gender discrimination in a veterinary setting were more likely to say that their experiences and considerations of gender discrimination had affected their career aspirations.

### Qualitative analysis

**FIGURE 5:**
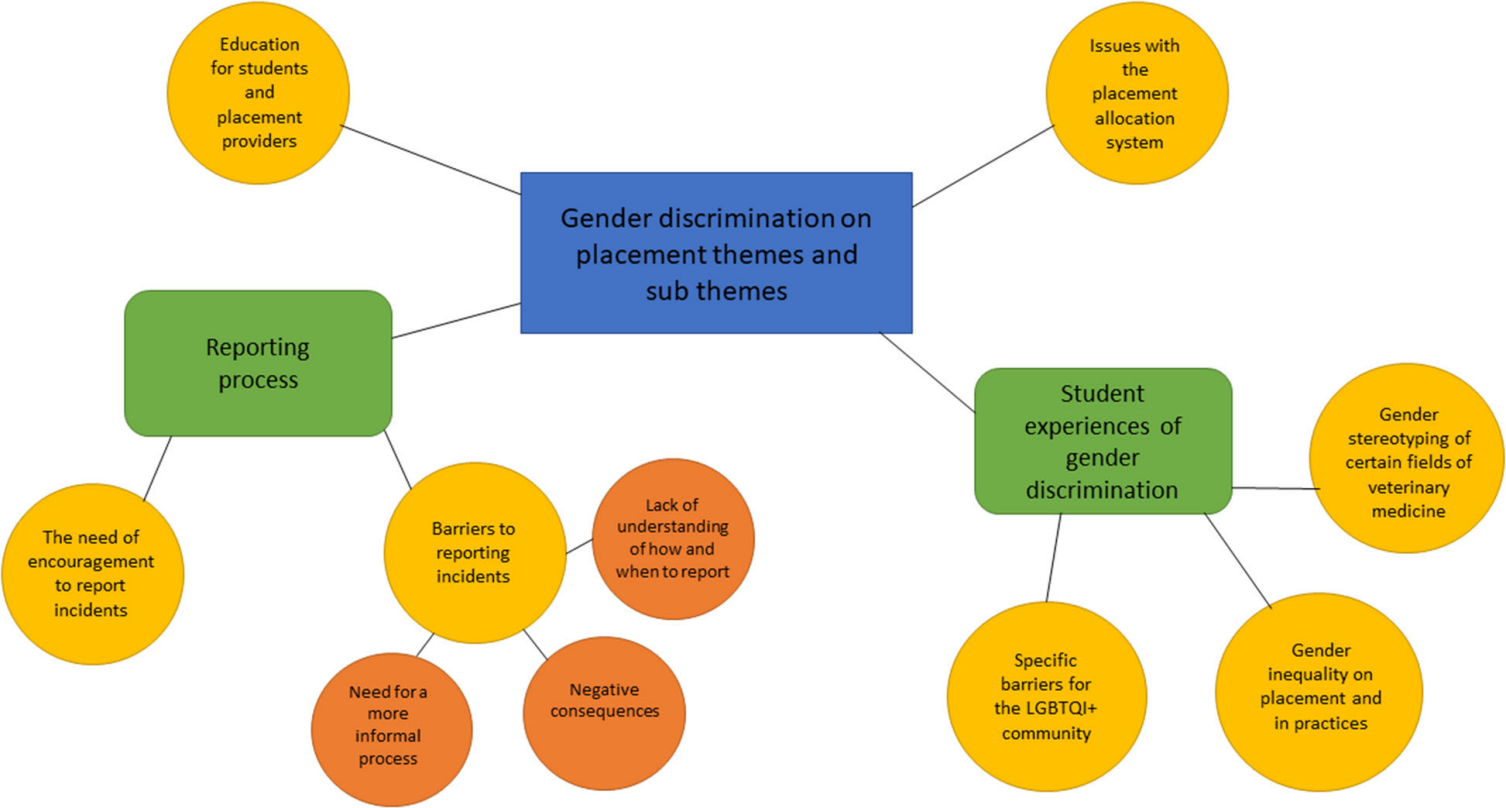
Qualitative themes and subthemes identified in focus groups of veterinary students on gender discrimination. LGBTQI+: lesbian, gay, bisexual, transgender, queer and intersex, plus

Three main themes emerged about student experiences of gender discrimination are as follows: (i) gender stereotyping of certain field of veterinary medicine, (ii) gender inequality on placements and in practices and (iii) specific barriers for the lesbian, gay, bisexual, transgender, queer and intersex, plus (LGBTQI+) community. Two main themes emerged about the reporting process include: (i) the need of encouragement to report incidents and (ii) barriers to reporting incidents. Two further themes of ‘education for students and placement providers’ and ‘issues with placement allocation system’ were also generated from the data (Figure 5).

The following is a series of representative quotes illustrating each of the themes described. The quotes provided are each followed by summary detail—origin, letter denotes what gender the student identifies as and number denotes year of study. For example, F4 means a student who identifies as female and who is in the fourth year of study. The quotes are organised by theme.

#### Gender stereotyping in certain fields of veterinary medicine

Analysis indicated that gender stereotyping exists within some fields of the veterinary profession. In particular, students reported that farm animal practice is perceived to be a masculine profession.

‘The main things that I have really been aware of is people saying that men are much more desirable as farm vets, find it easier to get a job in that field and that women are generally less desirable to employ as they go on maternity leave’. (Questionnaire, F4)

‘Even just saying that I'm doing veterinary medicine as a female then it's like they immediately presume I'd be going into like small animal veterinary, so I'm going to be looking after rabbits, but actually I'm really interested in farm animals’. (Focus groups, F1)

#### Gender inequality on placement and in practices

Students described situations in which they had experienced or witnessed gender inequality, they described incidents that occurred on placements, in practices and at university.

‘I definitely see it as identifying as male myself. I see that when I'm with students that identify as female on placement they are always treated like they can't do the stuff that I would be capable of doing, and the farmers would always say oh could you boys go to like lift the sawdust sacks or something and then to the girls you can go and feed the calves or something’. (Focus groups, M3)

#### Specific barriers for the LGBTQI+ community

Students described specific difficulties for members of the LGBTQI+ community.

‘I've found as a queer person like you don't want out yourself by saying something because you know there's all sorts of homophobic blather on a lot of farms and you don't want to be like well actually that's not OK’. (Focus groups, F5)

#### Ways of encouraging the reporting of incidents

Students suggested potential ways of increasing incident reporting, having the option for anonymity, a less formal process for reporting incidents and to have clear instructions for how to report incidents.

‘I think what the vet school can do is help people to feel confident about talking out, make it quite visible on the placement portal if there have been issues with the placement’. (Focus groups, F4)

#### Barriers to reporting incidents

Students described barriers stopping them from reporting incidents, including long‐term and short‐term consequences that may occur from reporting incidents. Concerns included repercussions from the host, not feeling as though incidents are taken seriously or properly dealt with and potentially having to make up weeks of missed EMS.

‘As a vet student you sometimes feel like you are in a weaker/inferior position to those discriminating against you—especially if those people are the ones giving your feedback or determining if you pass a rotation. You feel like you can't speak out and like it's not your place to do so’. (Questionnaire, F5)

‘Feeling like it's considered ‘normal’ so complaining won't change anything’. (Questionnaire, F5)

#### Education for students and placement providers

Students described the need for education—this centred around increased education for students on how to report incidents, how to deal with incidents themselves if appropriate and how to recognise discrimination. Students suggested the need for unconscious bias training for placement providers and staff.

‘I think just basically just talking about it more, obviously because in first year you have that kind of introduction to EMS talks …. And actually in every one of those talks just mention this (discrimination) is not OK. If you experience this, please contact us. The same as how they do like if you experience incident where you've hurt yourself or something’. (Focus groups, F4)

#### Issues with the placement allocation system

Students highlighted concerns about the placement allocation system. Students reported fears about the safety of going to unknown places alone and described incidents where they had discovered a placement in which they had a bad experience had received negative feedback on the placement portal, but this information was not shared.

‘When making the decision of where to undertake EMS placements, especially on farms where I might require accommodation I question whether I feel safe going on my own’. (Questionnaire, F1)

## DISCUSSION

### Student experiences of discrimination

Veterinary students reported experiencing gender discrimination most commonly on AHEMS placements. These mostly take place on farms and in rural settings, parallel to the gender discrimination reported by practicing veterinary surgeons, described most often in farm and mixed practice.^10^ On these placements, gender discrimination was most likely to come from a member of staff on placement or the student's placement supervisor. This may reflect a wider problem with the hierarchical dynamics of the profession that need to be addressed.^24^, ^25^, ^26^ The gender of the person discriminating was not collected in this survey, although work in other sectors identifies male and female academic staff were equally likely to discriminate.^27^

Gender stereotyping of farm animal practice has been reported previously.^28^, ^29^ It has been suggested that the male bias towards production animals medicine could be a legacy of the past.^28^ This contrasts with the findings of another study, which reported that these views still prevailed.^29^ A survey of 844 veterinary students in Australia^30^ found gender to have no effect on career intentions. It may be that UK culture is an influencing factor, or other factors outweigh social effects or biases in Australia. Traditional views of veterinary surgeons in the UK, as well as unconscious bias of respondents, may explain why the current study identified reasons for stereotyping farm practice as the perceived strength needed for farm animal work, or female students feeling they would not receive the same respect as male students. Some female students believed that maternity leave and having children would make them less employable, this was also found in medical students^31^ and qualified veterinary surgeons.^24^ Another study reported that some specialities of human medicine were gender stereotyped due to being perceived as ‘child friendly’.^32^ The perceived potential for injury and zoonotic illness for pregnant women when working with farm animals could explain why female students felt farm practice is male stereotyped.

The present study found that small animal practice was found to be gender stereotyped as female, this contrasts with the findings of a previous study, which reported that small animal practice was not gender stereotyped.^28^

### Barriers to reporting

Students identified a variety of barriers to reporting discrimination, including potential repercussions from the placement provider, their report not been adequately dealt with and a lack of understanding of how to report incidents. Under‐reporting is a common finding across studies including this study.^10^, ^33^, ^34^ Under‐reporting highlights the need for improved communication to students on the reporting process. Many of the barriers to reporting were similar to those found in studies of medical students^33^ and medical residents.^34^ One study reported that medical residents not reporting incidents thought the discrimination or harassment they experienced was harmless^34^; this was also stated by respondents in this study. This study suggests that there is a need to increase students’ awareness of what constitutes discrimination. While the focus of this study is on students’ experiences of discrimination and the actions they can take, the most effective way to reduce discrimination would be to reduce the incidence of discrimination directly. Steps should be taken to encourage those discriminating to adjust their behaviour alongside supporting students until society at large changes.

Since the term ‘reporting’ has negative connotations with students, more informal feedback processes such as those suggested in a previous study^32^ are recommended. For example, asking on a placement feedback form ‘did this individual treat you with respect?’ with a simple yes/no answer. Some students identified a stigma around reporting and suggested that more open discussion around discrimination would be helpful to reduce stigma. It was reported that some students had become ‘accultured’ to discrimination and believed that it was a normal thing to happen,^34^ this was supported by the results of our study.

The need for further education around gender equality and unconscious bias for staff and placement providers is supported by the results of this study. Researchers within a medical school in the USA found that implementing an intervention programme over 2 years drastically reduced the incidence of gender discrimination.^35^ A similar intervention within veterinary education is worth pursuing.

Student suggestions for reducing the incidence of gender discrimination on placements included education for students on dealing with incidents of discrimination themselves, education about recognising discrimination and reporting incidents. Researchers investigating nurses’ experiences found that they would like someone else to stand up for them if they were to experience discrimination, nurses also suggested that they would like to have classes on how to constructively correct someone if they were to experience discrimination.^36^ Inclusion of education for veterinary students on how to be an active bystander or an ally, and how to constructively correct someone could be of value to help students feel prepared to deal with discrimination in a veterinary setting or in everyday life.

Those students who identified as an underrepresented ethnic minority group were more likely to have experienced gender discrimination within a veterinary setting; this was also reported for medical students^33^ and practicing veterinary surgeons.^10^ Students who identified as being part of the LGBTQI+ community reported specific barriers while on placement, such as hiding their gender identity or sexuality and hearing homophobic and transphobic comments, this was consistent with a study of medical students and physicians.^37^ This shows the effects of intersectionality^25^; discrimination may have been against multiple different characteristics. Discrimination existing in this way suggests the need for further education for students, staff and placement providers about the diversity of students they will be working with; particularly those identifying as being within an ethnic minority group or those who are part of the LGBTQI+ community.

The results of this work may be affected by response and perception bias, due to the nature of the topic area and because incidents of discrimination were self‐described within the questionnaire and focus groups. The use of the Likert scale with the midpoint option of ‘no opinion either way’ could be interpreted in multiple ways. However, the exact terminology used has previously been found to make negligible difference,^38^ it was felt that this did not impact the overall conclusions of this study. It is possible that further information could have been garnered from the use of a numbered scale for questions regarding methods to decrease incidents and the impact on career aspirations. Further work investigating effective methods of decreasing incidents of discrimination, with a study population including students studying veterinary medicine at other institutions would be beneficial. For this study, all non‐white ethnic groups were analysed together to maintain anonymity due to small sample sizes. While the demographics represent those within UK veterinary schools more widely, students of different ethnic groups experience discrimination differently. This could also perpetuate the feeling of white versus other. The results of this study suggest that further research is needed to investigate discrimination experienced by students who identify as LGBTQI+. It is difficult to effectively study one type of discrimination in isolation of others. Discrimination may have been against multiple different characteristics, yet this intersectionality may not have been captured in this study.

In summary, students experienced gender discrimination within veterinary and farm settings, predominantly female students on AHEMS placements. This discrimination impacted on students’ career aspirations and may contribute to recruitment and retention challenges in the profession. Students are reluctant to report incidents for a variety of reasons and some perceived the reporting process as ineffective. Therefore, effective and supportive reporting mechanisms are needed more than ever. Educating students on how to recognise and report gender discrimination is necessary, as it empowers them to deal with discrimination themselves. Albeit, only with education for all university staff and placement providers can we foster an inclusive environment for everyone.

Solely addressing the gender inequality within the veterinary profession will not be enough to make the profession fully inclusive. The inequality and discrimination against other protected and non‐protected characteristics need to be addressed to improve the inclusivity of diversity of the veterinary profession.

## AUTHOR CONTRIBUTIONS

All authors contributed to the planning, execution and writing of this manuscript. Katie Freestone performed data collection and analysis and wrote the original draft of the manuscript. John Remnant and Erica Gummery supervised the project and reviewed and edited the manuscript.

## CONFLICTS OF INTEREST

The authors declare they have no conflicts of interest.

## FUNDING INFORMATION

The authors received no specific funding for this work.

## ETHICS STATEMENT

Ethical approval was granted from the University of Nottingham, School of Veterinary Medicine and Science (reference 3198‐200706).

## Supporting information



Veterinary student's experiences and perceptions of gender discrimination

Supplementary material: Summary of responses to a survey of undergraduate veterinary students at the University of Nottingham School of Veterinary Medicine and Science

## Data Availability

Research data are not shared.
