# Supplementary material for: Gender discrimination of veterinary students and its impact on career aspiration: A mixed methods approach
Source: Vet Rec Open. 2022 Nov 1;9(1):e47. doi: 10.1002/vro2.47 (PMC9624077; doi:10.1002/vro2.47)
Supplement: Supplementary file 1 — Veterinary student's experiences and perceptions of gender discrimination [file VRO2-9-e47-s001.pdf]

Veterinary student's experiences and perceptions of gender discrimination

Page 1: Introduction

Introduction

Researchers

Katie Freestone

Erica Gummery

John Remnant

Kate Cobb

Purpose of Study

To find out veterinary students perceptions and experiences of gender discrimination and whether it affects their career aspirations. The findings could be used to inform teaching on gender equality.

Consent

This consent form is a way of indicating that you agree to take part in this study and that you understand any information collected:

- Will be used for a research study
- May be written in a report for publication
- May be presented at research conferences or meetings
- Will be anonymised and treated confidentially
- Will only be accessed by research colleagues or examiners
- Will be stored and destroyed according to current data protection regulations GDPR

All questions are optional and you may stop at any time.

Disclaimer

This questionnaire is not a way of reporting an incident of gender discrimination. If you would like to report an incident of discrimination that has taken place please contact the placements team, welfare team, your tutor or a trusted member of staff.

Veterinary-Placement@exmail.nottingham.ac.uk

SV-Senior-Tutor@exmail.nottingham.ac.uk

SS-Welfare-SB@exmail.nottingham.ac.uk

Page 2: Demographics

These questions are to collect demographic information and see if differences in demographics affects experiences and perceptions of gender discrimination.

This data will be anonymised and where there are few individuals in a category the data will be presented in an aggregated form so that individuals cannot be identified.

1. With which gender do you most identify?

Please select no more than 1 answer(s).

- ☐ Male
- ☐ Female
- ☐ Other gender identity
- ☐ Prefer not to say

1.a. If you selected Other gender identity, please specify:

____

2. Which vet school do you currently attend?

Please select no more than 1 answer(s).

- ☐ Nottingham
- ☐ Bristol
- ☐ Surrey
- ☐ Liverpool
- ☐ RVC
- ☐ Cambridge
- ☐ Edinburgh

- [ ] Glasgow

3. What is your current year of study?

Please select no more than 1 answer(s).

- [ ] Prelim/Gateway
- [ ] 1
- [ ] 2
- [ ] 3
- [ ] 4
- [ ] 5
- [ ] 6 (Cambridge only)
- [ ] Intercalating
- [ ] Other

3.a. If you selected Other, please specify:

4. What is your age?

5. With which ethnic group do you most identify?

Please select no more than 1 answer(s).

- [ ] Asian or Asian British- Bangladeshi

- ☐ Asian or Asian British- Pakistani
- ☐ Asian or Asian British- Indian
- ☐ Asian or Asian British- Any other Asian background (please specify)
- ☐ Black or Black British- African
- ☐ Black or Black British- Caribbean
- ☐ Black or Black British- Any other Black background
- ☐ Chinese or other Asian ethnic group- Chinese
- ☐ Chinese or other Asian ethnic group- Any other Asian ethnic background (please specify)
- ☐ Mixed- White and Asian
- ☐ Mixed- White and Black African
- ☐ Mixed- White and Black Caribbean
- ☐ Mixed- any other Mixed background (please specify)
- ☐ White- British
- ☐ White- Irish
- ☐ White- Gypsy or Traveller
- ☐ White- any other White background (please specify)
- ☐ Prefer not to say
- ☐ Other

5.a. If you selected Other, please specify:

____

6. What are the 1st 3 or 4 digits of your home post code (Eg. LE14 or DN2) (ie.the postcode of where you grew up)? *Optional*

____

Page 3: Career Aspirations

7. In which field(s) of veterinary do you most aspire to work within?

- ☐ Not sure
- ☐ Small animal practice
- ☐ Mixed practice
- ☐ Farm practice
- ☐ Equine practice
- ☐ Exotic practice/ Zoo
- ☐ Research/ Academia
- ☐ Education
- ☐ Government
- ☐ Other

7.a. If you selected Other, please specify:

Page 4: Gender discrimination

These questions are to identify student's perceptions and experiences of gender discrimination.

Gender discrimination- Any action that specifically denies opportunities, privileges or rewards to a person because of their gender. (Kristoffersson et al., 2016)

Forms of gender discrimination

- Verbal harassment- for example someone saying there is no point promoting women as they will go off and have children.
- Physical sexual harassment- for example touching inappropriately.
- Verbal sexual harassment- for example a university lecturer making sexual jokes to one of their students and implying that the student will pass their exams if they sleep them.
- Direct discrimination- for example nightclub allowing women free entry but charging men to enter.

Forms of discrimination and examples taken from the equality and human rights commission. https://www.equalityhumanrights.com/en/advice-and-guidance/sex-discrimination

8. To what extent do you agree with the following statements:

	Strongly disagree	Disagree	No opinion either way	Agree	Strongly agree
Gender discrimination is present within the veterinary profession.	☐	☐	☐	☐	☐
I am concerned about gender discrimination within the veterinary profession.	☐	☐	☐	☐	☐

All veterinary students have equal opportunities to reach their goals/ aims on EMS regardless of their gender.	☐	☐	☐	☐	☐
Discrimination against my gender has negatively affected my experiences on EMS.	☐	☐	☐	☐	☐
My gender has not limited my opportunities on EMS.	☐	☐	☐	☐	☐

9. Have you personally experienced gender discrimination whilst in a veterinary setting (eg. On EMS, at vet school)?

Please select no more than 1 answer(s).

- ☐ Yes
- ☐ No

9.a. Where did you experience it? (if multiple incidences select all that apply)

- ☐ Vet School
- ☐ On Animal Husbandry EMS (AHEMS)
- ☐ Clinical EMS (CEMS): Small animal
- ☐ CEMS: Mixed
- ☐ CEMS: Equine
- ☐ CEMS: Farm
- ☐ CEMS: Laboratory/ research facility
- ☐ CEMS: Exotic
- ☐ Other

9.a.i. If you selected Other, please specify:

9.b. Did you take action (eg. report it, say something back)?

- [ ] Never
- [ ] Sometimes
- [ ] Every time

9.c. Who was the person discriminating? (if multiple incidences select all that apply)

- [ ] Placement supervisor
- [ ] Another member of placement staff
- [ ] Client
- [ ] Another student
- [ ] A member of university staff
- [ ] Other

9.c.i. If you selected Other, please specify:

9.d. What form of gender discrimination was it? (if multiple incidences select all that apply)(see top of page for descriptions)

- [ ] Verbal harrassment
- [ ] Physical sexual harrassment
- [ ] Verbal sexual harrassment
- [ ] Direct discrimination
- [ ] Prefer not to say
- [ ] Other

9.d.i. If you selected Other, please specify:

10. Have you witnessed students (other than yourself) experiencing gender discrimination in a veterinary setting?

- Yes
- No

10.a. Where did you witness it? (if multiple incidences select all that apply)

- Vet School
- Animal Husbandry EMS (AHEMS)
- Clinical EMS (CEMS): Small Animal
- CEMS: Mixed
- CEMS: Farm
- CEMS: Equine
- CEMS: Exotics
- CEMS: Laboratory/ research facility
- Other

10.a.i. If you selected Other, please specify:

10.b. Did you take any action (eg. report it, say something back)?

- Never
- Sometimes

- Every time

11. Have you heard about students experiencing gender discrimination within a veterinary setting?

- [ ] Yes
- [ ] No

11.a. Where did they experience it? (if multiple incidences select all that apply)

- [ ] Don't know
- [ ] Vet School
- [ ] On Animal Husbandry EMS (AHEMS)
- [ ] Clinical EMS (CEMS): Small animal
- [ ] CEMS: Mixed
- [ ] CEMS: Equine
- [ ] CEMS: Farm
- [ ] CEMS: Exotics
- [ ] CEMS: Laboratory/ Research facility
- [ ] Other

11.a.i. If you selected Other, please specify:

12. If you didn't report an incident of gender discrimination what was your main reason for not doing so?

13. To what extent do you agree with the following statement?: If I were to experience gender discrimination in the future I would report it.

Please select no more than 1 answer(s).

- ☐ Yes
- ☐ No

14. To what extent do you agree with the following statement?: If I were to report an incident of gender discrimination appropriate action would be taken.

Please select no more than 1 answer(s).

- ☐ Strongly disagree
- ☐ Disagree
- ☐ Agree
- ☐ Strongly agree

15. Do you think any of the following would be useful to decrease the incidence of gender discrimination?

- ☐ Clear guidance on who to report an incident to
- ☐ Awareness of university policies on gender discrimination
- ☐ Teaching on gender equality
- ☐ Other

15.a. If you selected Other, please specify:

16. To what extent have your considerations and experiences of gender discrimination affected your career aspirations (eg. choice of species, aspirations to take on a leadership role)?

Please select no more than 1 answer(s).

- ☐ Not at all
- ☐ A little
- ☐ A lot
- ☐ The deciding factor

17. If you have any further comments please write them here:

Page 5: Prize draw and focus groups

18. If you would like to be entered for a random draw to win a £50 Amazon voucher or be contacted to take part in focus groups please leave your email address below (your email adress will be separated from your survey responses):

19. Please indicate your preferences

- ☐ I would like to be entered in the prize draw
- ☐ I am willing to be contacted about taking part in focus groups for the project

Page 6: Final page

Thank you for taking part in this questionnaire!

For further support about any of the topics raised in this questionnaire, don't hesitate to contact any of the following:

- The university placements team, senior tutors or welfare team:

  Veterinary-Placement@exmail.nottingham.ac.uk

  SV-Senior-Tutor@exmail.nottingham.ac.uk

  SS-Welfare-SB@exmail.nottingham.ac.uk

- Vetlife https://www.vetlife.org.uk/ Phone: 03030402551
- Mind https://www.mind.org.uk/

If you have any questions about this project please contact Katie Freestone svykf@nottingham.ac.uk.
