# Supplementary material for: Gender discrimination of veterinary students and its impact on career aspiration: A mixed methods approach
Source: Vet Rec Open. 2022 Nov 1;9(1):e47. doi: 10.1002/vro2.47 (PMC9624077; doi:10.1002/vro2.47)
Supplement: Supplementary file 2 — Supplementary material: Summary of responses to a survey of undergraduate veterinary students at the University of Nottingham School of Veterinary Medicine and Science [file VRO2-9-e47-s002.pdf]

Supplementary material: Summary of responses to a survey of undergraduate veterinary students at the University of Nottingham School of Veterinary Medicine and Science

**Characteristic**	**N = 261**[1]
With which gender do you most identify?	
Female	217 (83%)
Male	42 (16%)
Other gender identity	2 (0.8%)
What is your current year of study?	
Preliminary	4 (1.5%)
1	90 (34%)
2	44 (17%)
3	47 (18%)
4	35 (13%)
5	41 (16%)
What is your age?	20 (19, 22)
No response	1
With which ethnic group do you most identify?	

Characteristic	N = 261[1]
Asian or Asian British- Any other Asian background (please specify)	1 (0.4%)
Asian or Asian British- Bangladeshi	1 (0.4%)
Asian or Asian British- Indian	4 (1.5%)
Black or Black British- African	1 (0.4%)
Black or Black British- Caribbean	3 (1.1%)
Chinese or other Asian ethnic group- Chinese	2 (0.8%)
Mixed- any other Mixed background (please specify)	2 (0.8%)
Mixed- White and Asian	6 (2.3%)
Mixed- White and Black African	1 (0.4%)
Prefer not to say	2 (0.8%)
White- any other White background (please specify)	17 (6.5%)
White- British	214 (82%)
White- Irish	7 (2.7%)
In which field(s) of veterinary do you most aspire to work within?	(Multiple options)
Education	21 (8.0%)

Characteristic	N = 261[1]
Equine practice	55 (21%)
Exotic practice/ Zoo	68 (26%)
Farm practice	68 (26%)
Government	6 (2.3%)
Mixed practice	91 (35%)
Not sure	36 (14%)
Other	12 (4.6%)
Research/ Academia	14 (5.4%)
Small animal practice	118 (45%)
Gender discrimination is present within the veterinary profession.	
Strongly agree	35 (13%)
Agree	159 (61%)
No opinion either way	39 (15%)
Disagree	25 (9.6%)
Strongly disagree	3 (1.1%)

**Characteristic**	**N = 261**[1]
I am concerned about gender discrimination within the veterinary profession.	
Strongly agree	19 (7.3%)
Agree	107 (41%)
No opinion either way	68 (26%)
Disagree	59 (23%)
Strongly disagree	8 (3.1%)
All veterinary students have equal opportunities to reach their goals/ aims on EMS regardless of their gender.	
Strongly agree	35 (13%)
Agree	75 (29%)
No opinion either way	45 (17%)
Disagree	92 (35%)
Strongly disagree	14 (5.4%)
Discrimination against my gender has negatively affected my experiences on EMS.	
Strongly agree	10 (3.8%)

Characteristic	N = 261[1]
Agree	47 (18%)
No opinion either way	63 (24%)
Disagree	79 (30%)
Strongly disagree	62 (24%)
My gender has not limited my opportunities on EMS	
Strongly agree	67 (26%)
Agree	83 (32%)
No opinion either way	59 (23%)
Disagree	42 (16%)
Strongly disagree	9 (3.5%)
No response	1
Have you personally experienced gender discrimination whilst in a veterinary setting (eg. On EMS, at vet school)?	Yes: 88 (34%)
No response	1
Where did you experience it? (if multiple incidences select all that apply)	(Multiple answers)
CEMS: Equine	5 (1.9%)

Characteristic	N = 261[1]
CEMS: Farm	7 (2.7%)
CEMS: Mixed	5 (1.9%)
Clinical EMS (CEMS): Small animal	8 (3.1%)
On Animal Husbandry EMS (AHEMS)	68 (26%)
Other	10 (3.8%)
Vet School	7 (2.7%)
Did you take action (eg. report it, say something back)?	
Never	56 (64%)
Never,Sometimes	1 (1.1%)
Sometimes	26 (30%)
Every time	5 (5.7%)
No response	173
Who was the person discriminating? (if multiple incidences select all that apply)	Multiple answers
A member of university staff	4 (1.5%)
Another member of placement staff	50 (19 %)

Characteristic	N = 261[1]
Another student	7 (2.7%)
Client	26 (10%)
Other	5 (1.9%)
Placement supervisor	28 (11%)
What form of gender discrimination was it? (if multiple incidences select all that apply)	(Multiple answers)
Direct discrimination	26 (10%)
Other	9 (3.4%)
Physical sexual harrassment	9 (3.4%)
Verbal harrassment	63 (24%)
Verbal sexual harrassment	22 (8.4%)
Have you witnessed students (other than yourself) experiencing gender discrimination in a veterinary setting?	
No	204 (79%)
Yes	52 (20%)
Yes,No	2 (0.8%)
No response	3

Characteristic	N = 261[1]
Where did you witness it? (if multiple incidences select all that apply)	(Multiple Answers)
Animal Husbandry EMS (AHEMS)	43 (16%)
CEMS: Equine	2 (0.77%)
CEMS: Farm	3 (1.1%)
Clinical EMS (CEMS): Small Animal	9 (3.4%)
Other	3 (1.1%)
Vet School	9 (3.4%)
Did you take any action (eg. report it, say something back)?	
Never	30 (57%)
Sometimes	20 (38%)
Every time	3 (5.7%)
No response	208
Have you heard about students experiencing gender discrimination within a veterinary setting?	
No	92 (35%)
Yes	165 (63%)

**Characteristic**	**N = 261**[1]
Yes,No	3 (1.2%)
No response	1
Where did they experience it? (if multiple incidences select all that apply)	
CEMS: Equine	14 (5.4%)
CEMS: Exotics	1 (0.38%)
CEMS: Farm	46 (18%)
CEMS: Laboratory/ Research facility	1 (0.38%)
CEMS: Mixed	10 (3.8%)
Clinical EMS (CEMS): Small animal	24 (9.2%)
Don't know	25 (9.6%)
On Animal Husbandry EMS (AHEMS)	117 (45%)
Other	2 (0.77%)
Vet School	17 (6.5%)
Do you agree with the following statement?: If I were to experience gender discrimination in the future I would report it.	Yes: 166 (64%)

**Characteristic**	**N = 261**[1]
To what extent do you agree with the following statement?: If I were to report an incident of gender discrimination appropriate action would be taken.	
Strongly agree	9 (3.4%)
Agree	133 (51%)
Disagree	107 (41%)
Strongly disagree	12 (4.6%)
Select any of the following you think would be useful to decrease the incidence of gender discrimination?	
Awareness of university policies on gender discrimination	133 (51%)
Clear guidance on who to report an incident to	208 (80%)
Other	22 (8.4%)
Teaching on gender equality	130 (50%)
To what extent have your considerations and experiences of gender discrimination affected your career aspirations (eg. choice of species, aspirations to take on a leadership role)?	
Not at all	134 (51%)
A little	111 (43%)
A lot	15 (5.7%)

Characteristic	N = 261[1]
The deciding factor	1 (0.4%)

[1] Median (IQR); n (%)
